# The Awareness and Knowledge of Normal and Abnormal Signs and Symptoms of Pregnancy Among Women of Childbearing Age in Saudi Arabia

**DOI:** 10.7759/cureus.44470

**Published:** 2023-08-31

**Authors:** Noof M Alharbi, Shatha A Al Zahrani, Taghreed H Basri, Sundus A Tawfiq, Nawal Y Jokhdar, Raghad Y AlQahtani, Haneen S Khair, Noor A Alghanem

**Affiliations:** 1 College of Medicine, King Saud bin Abdulaziz University for Health Sciences, Riyadh, SAU; 2 College of Nursing, Al Baha University, Al Baha, SAU; 3 Department of Obstetrics and Gynecology, Madinah Health Cluster, Ohud Hospital, Madinah, SAU; 4 College of Medicine, Taibah University, Medina, SAU; 5 College of Medicine, Ibn Sina National College for Medical Studies, Jeddah, SAU; 6 Department of Surgery, King Khalid University, Abha, SAU; 7 Department of General Practice, Dar Al-Uloom University, Riyadh, SAU

**Keywords:** symptoms, signs, saudi arabia, pregnancy, knowledge, awareness

## Abstract

Background: Pregnancy is a natural physiological process that leads to the outstanding outcome of giving birth. It involves normal and abnormal signs and symptoms that women need to be aware of during this period.

Objectives: This study aims to assess the awareness and knowledge about normal and abnormal signs and symptoms of pregnancy among women of childbearing age in Saudi Arabia.

Methods: This is an online questionnaire-based cross-sectional study done in 2023 among women in Saudi Arabia, aged 18 to 45. The estimated sample size was 385 after applying inclusion and exclusion criteria. However, 981 responses were collected and included.

Results: A total of 981 participants were included in our study. The study findings noted that the majority of women demonstrated a good level of knowledge about the normal signs and symptoms of pregnancy. About 82% of the women reported that they knew mood swings were normal symptoms during pregnancy, 80.1% were aware of nausea, 75.9% knew about fatigue, and 68.9% knew about fainting or dizziness. The findings noted that less than half of the participants were fully aware of the abnormal obstetric signs. For the abnormal signs and symptoms during the first trimester, only 45.2% were fully aware of the symptoms. In the second trimester, 39.0% were fully aware of the symptoms. Only 30.5% of the participants were fully aware of the abnormal signs and symptoms in the third trimester. The study established a statistically significant relationship between age and knowledge of abnormal symptoms during the first, second, and third trimesters (P=0.027, 0.041, and 0.006) (r=0.139, 0.105, and 0.146). Furthermore, a statistically significant relationship was found between the level of education of the participants and the level of awareness of abnormal symptoms during the first trimester (P=0.043). However, there was no significant relationship between education level and the level of knowledge regarding abnormal symptoms during the second and third trimesters (r=0.22, 0.061, P=0.578, 0.603).

Conclusion: The study found that the majority of women had a good level of awareness and knowledge about normal signs and symptoms of pregnancy, while less than half of the women were fully aware of the abnormal signs and symptoms at different stages of pregnancy. The older respondents and people with higher levels of education demonstrated more knowledge. The two variables, age and education level, had a statistically significant relationship with knowledge of abnormal signs and symptoms of pregnancy with education level being only significant during the first trimester period. Our study concluded that women had different symptoms during pregnancy, both normal and abnormal. Their knowledge about these signs and symptoms was considerably moderate, but further awareness about the normality of these signs is needed. The study recommends more research to measure women's awareness about normal and abnormal symptoms of pregnancy, and more awareness programs should be carried out in the region as a way of enhancing a better understanding of normal and abnormal pregnancy symptoms. This will go a long way in helping women through their pregnancy and make them more comfortable.

## Introduction

Every woman has a different experience during pregnancy with wide variation in signs, symptoms, and physiological changes [1]. Significant physical and psychological changes occur throughout pregnancy. These changes influence the quality of life of pregnant women and have an impact on the health of both the mother and her child [2]. Theoretically, women can conceive from the time they have their monthly cycles, known as menarche, during adolescence until they reach menopause, which is the end of the monthly cycle. This time frame is known as the “childbearing age,” with menarche as the start and menopause as the end. The average woman's reproductive years last from 12 to 51 years [3]. Hormonal, physical, and psychological changes during pregnancy contribute to the variation in the signs and symptoms among women. Early warning signs include fatigue, tender breasts, nausea with or without vomiting, increased urination, and changes in bowel habits like constipation, abdominal bloating, and light spotting. Cramping may also occur. The earliest and most reliable sign is a missed period [4]. When it comes to the gestational period, a singleton pregnancy typically lasts 40 weeks (280 days) from the first day of the last period to the scheduled delivery date. Previously, “term” was referred to the timeframe that started three weeks before the planned delivery date and ended two weeks later. This was with the goal of consistent and positive newborn outcomes from deliveries over this time period [5]. The high level of knowledge about the signs and symptoms of pregnancy has a notable effect on increasing women's interest in their health during the prenatal period and increasing their commitment to attending prenatal clinics. About 85% of women who are pregnant have consideration about pregnancy-related symptoms during the preventive health checks performed by their GP or midwife [6]. Health records promote a biomedical focus on serious health issues, while symptoms, such as nausea, are given less attention as they may be present in otherwise normal pregnancies. Pregnancy-related physical symptoms are common throughout the first trimester [7]. Nausea is reported by approximately 80% of women, while vomiting affects 35-40% throughout the first trimester [8,9]. Frequencies in the order of 25-50% have also been described for symptoms such as vulvar itching, vaginal bleeding, and pelvic cavity pain [10]. Pelvic girdle pain and low back pain symptoms (or, in some studies, a combination of these two referred to as "lumbopelvic pain") are often reported for all three trimesters, with a prevalence of 20-65% [11]. It's important to note that some symptoms might indicate a pathological pregnancy, but most are physiological changes that normally occur as a result of pregnancy [12]. As the harm of these symptoms is not well understood, physicians may be ignorant of the concerns of their pregnant patients. It has only been studied in a few studies how women feel about their pregnancy-related physical symptoms [13,14]. It has been reported that various depression and anxiety measuring instruments, such as the Cambridge Concern Scale, have been used to assess maternal stress or worries during pregnancy, but these measures are global in nature and do not address the impact of specific symptoms [15]. This study aims to assess knowledge about normal and abnormal signs and symptoms of pregnancy among women of childbearing age in Saudi Arabia.

## Materials and methods

This is a cross-sectional study that was conducted in Saudi Arabia in 2023. The online questionnaires were distributed via social media including Twitter, WhatsApp, and Telegram to collect data. The target population was women between the ages of 18 and 45 in all regions of Saudi Arabia. The inclusion criteria were women aged between 18 and 45 who have at one point in time been pregnant, in Saudi Arabia, Arabic speakers, and residents of Saudi Arabia. The exclusion criteria were the minors (less than 18 years) who had never been pregnant and those who declined to participate in the study. The sample size was calculated based on an infinite population and estimated as 50%. Using a 95% confidence interval and a 5% margin of error, the calculated sample size was 385. The sample size was calculated using a Raosoft calculator (Raosoft, Inc., Seattle, WA 98115, United States). However, the study involved 981 participants who completed the online questionnaires.

The questionnaire comprised of sociodemographic and obstetrical characteristics of participants. These attributes included the participants’ living region, nationality, age, level of education, employment status, economic status, and smoking status. Other attributes were comorbidities, blood group, number of pregnancies, number of miscarriages, gestational age, number of children, signs of pregnancy in the first, second, and third trimesters, and the use of folic acid supplements. Additionally, the questionnaire contained 42 focused questions developed in English and translated into Arabic to determine the participants’ level of awareness about normal signs and symptoms of pregnancy as well as the abnormal symptoms during the first, second, and third trimesters of pregnancy.

Data input and analysis were performed using SPSS Statistics version 23.0 (IBM Corp. Released 2015. IBM SPSS Statistics for Windows, Version 23.0. Armonk, NY: IBM Corp.). Frequency and percentage are shown for categorical data. To see if there are any correlations between qualitative variables, the correlation test was used. Using a test, quantitative variables were analyzed. Statistical significance is commonly defined as a p-value of less than 0.05. The data was kept private, and only the PI and co-authors had access to it.

This research did not receive any specific grant from funding agencies in the public, commercial, or not-for-profit sectors. The study has been approved by the research ethics committee at the General Directorate of Health Affairs in Madinah with the approval number H-03-M-84.

## Results

A total of 981 participants were able to complete the online questionnaires. Most of them were within the age group of 36-45 years (35.8%). A large percentage of participants (88.3%) were Saudi nationals; more than two-thirds of the participants were married at the time of the study (69.4%), and 71.1% had a bachelor’s degree. Regarding employment status, 43.6% were unemployed/housewives and 31.8% were employed. Their economic status was as follows: <5000 SR (65.9%), 5000-10000 (19.6%), and 10000-15000 (15.9%). About 10.6% of the participants were smokers (Table 1).

**Table 1:** Demographic characteristics of the study participants (n=981)

Variable	Categories	Frequency	Percent
Age (in years)	18-25	313	31.9
	26-35	317	32.3
	36-45	351	35.8
Nationality	Saudi	866	88.3
	Non-Saudi	115	11.7
Marital status	Single	247	25.2
	Married	681	69.4
	Divorced	39	4
	Widowed	14	1.4
Educational level	Primary	18	1.8
	Intermediate	29	3
	Secondary	168	17.1
	Bachelor’s degree	688	70.1
	Postgraduate	78	8
Employment status	Employed	312	31.8
	Unemployed/housewife	428	43.6
	Student	238	24.3
	Retired	3	0.3
Economic status (SAR)	<5,000	558	56.9
	5,000-10,000	192	19.6
	10,000-15,000	156	15.9
	>15,000	75	7.6
Smoking status	Smoker	104	10.6
	Non-smoker	840	85.6
	Ex-smoker	37	3.8

In terms of clinical characteristics of the participants, 79.9% reported that they didn`t have chronic diseases, 31.8% were O+ blood grouping, and 28.5% were A+. About the number of their children, 42.7% of the participants reported that they had one to three children, 33.3% had none, and 22% had four to six children. About 27.7% were pregnant two to three times, and 21.3% were pregnant four to five times. Regarding abortion history, 73.2% never had it, and 22.9% had it less than three times. About 88% of them were not pregnant at the time of the study, 49.7% knew they were pregnant by using a home pregnancy test, and 24.9% had never been pregnant. About 60.8% of the respondents used a folic acid supplement, and 32.6% didn`t (Table 2).

**Table 2:** Medical, surgical, and obstetrical history

Variable	Categories	Frequency	Percent
Chronic diseases	Hypertension	50	5.1
	Diabetes mellitus	18	1.8
	Asthma	31	3.2
	Thyroid problems	64	6.5
	None	784	79.9
	Others	34	3.5
Blood group	AB+	34	3.5
	A+	280	28.5
	B+	133	13.6
	O+	312	31.8
	A-	36	3.7
	B-	28	2.9
	O-	64	6.5
	AB-	9	0.9
	I don’t know	85	8.7
No. of children	None	327	33.3
	1-3	419	42.7
	4-6	216	22
	7 or more	19	1.9
No. of pregnancies	None	276	28.1
	Primigravida	132	13.5
	2-3 times	272	27.7
	4-5 times	209	21.3
	≥6 times	92	9.4
No. of abortions	None	718	73.2
	Less than three times	225	22.9
	≥3 times	38	3.9
Gestational age	Not pregnant	863	88
	First trimester	39	4
	Second trimester	50	5
	Third trimester	29	3
If you are pregnant or have you ever been, how did you know?	Experience from a previous pregnancy	55	5.6
	By ultrasound in the hospital	17	1.7
	By laboratory pregnancy test	177	18
	By home pregnancy test	488	49.7
	I have never been pregnant	244	24.9
Do you use a folic acid supplement?	Yes	596	60.8
	No	385	39.2

Regarding the knowledge of normal pregnancy symptoms, 82% of the participants reported that they knew that mood swings were a normal symptom during pregnancy, 80.1% were aware of nausea, 75.9% knew about fatigue, 68.9% fainting or dizziness, 67.4% and 67.1% knew of heartburn and vomiting, respectively, and 65.9% knew cravings or an extreme aversion to eating and vaginal discharge with no itching. Only 25.9% of the respondents were aware that those symptoms still persist (Table 3).

**Table 3:** Knowledge of normal pregnancy symptoms

Symptoms	Yes	No
Fainting/dizziness	676 (68.9)	305 (31.1)
Nausea	786 (80.1)	195 (19.9)
Vomiting	658 (67.1)	323 (32.9)
Fatigue	745 (75.9)	236 (24.1)
Heartburn	661 (67.4)	320 (32.6)
Mood swings	804 (82)	177 (18)
Cravings or extreme aversion to eating	646 (65.9)	335 (34.1)
Swelling or pain in the breast	574 (58.5)	407 (41.5)
Constipation	533 (54.3)	448 (45.7)
Vaginal discharge with no itching	646 (65.9)	335 (34.1)
Abdominal cramp	636 (64.8)	345 (35.2)
Do these symptoms still persist?	254 (25.9)	727 (74.1)

Considering the awareness of abnormal signs and symptoms occurring during different stages of pregnancy including the first, second, and third trimesters, persistent mood swings were the most known abnormal symptoms (82%), followed by persistent nausea (80.1%) and severe fatigue (75.9%). During the first trimester of pregnancy, persistent mood swings and persistent nausea were the most known abnormal symptoms. During the second trimester, back pain (65.5%) was the top-mentioned symptom followed by stretch marks in the skin (59.8%) and then headache (48.9%). During the third trimester, frequent urination (65.4%) was the most known symptom followed by difficulty sleeping (57.2%) and lower limb edema (49%) (Table 4).

**Table 4:** Proportion of women who reported knowledge of abnormal symptoms in the first, second, and third trimesters

Knowledge of abnormal symptoms in the first trimester	Yes	No
Persistent fainting/dizziness	676 (68.9)	305 (31.1)
Persistent nausea	786 (80.1)	195 (19.9)
Severe vomiting	658 (67.1)	323 (32.9)
Severe fatigue	745 (75.9)	236 (24.1)
Persistent heartburn	661 (67.4)	320 (32.6)
Persistent mood swings	804 (82)	177 (18)
Extreme aversion to eating	646 (65.9)	335 (34.1)
Severe swelling or pain in the breast	574 (58.5)	407 (41.5)
Severe constipation	533 (54.3)	448 (45.7)
Abnormal vaginal discharge with no itching	646 (65.9)	335 (34.1)
Severe abdominal cramp	636 (64.8)	345 (35.2)
Knowledge of abnormal symptoms in the second trimester	Yes	No
Oesophageal reflux	402 (41)	579 (59)
Stretch marks on the skin	587 (59.8)	394 (40.2)
Enlarged breasts	469 (47.8)	512 (52.2)
Beast pain	424 (43.2)	557 (56.8)
Swollen or bleeding gums	227 (23.1)	754 (76.9)
Bleeding from the nose	99 (10.1)	882 (89.9)
Pain in a specific place around the abdomen	324 (33)	657 (67)
Headache	480 (48.9)	501 (51.1)
Blurred vision	267 (27.2)	714 (72.8)
Back pain	643 (65.5)	338 (34.5)
Muscle spasm in the bottom of the foot	407 (41.5)	574 (58.5)
Fever	374 (38.1)	607 (61.9)
Knowledge of abnormal symptoms in third trimester	Yes	No
Lower limbs edema	481 (49)	500 (51)
Severe abdominal pain	315 (32.1)	666 (67.9)
Brown discharge	195 (19.9)	786 (80.1)
False contractions	466 (47.5)	515 (52.5)
False divorce	373 (38)	608 (62)
Piles	276 (28.1)	705 (71.9)
Varicose veins	252 (25.7)	729 (74.3)
Frequent urination	642 (65.4)	339 (34.6)
Urinary incontinence	147 (15)	834 (85)
Rapid weight gain	437 (44.5)	544 (55.5)
Constipation	395 (40.3)	586 (59.7)
Shortness of breath or difficulty breathing	438 (44.6)	543 (55.4)
Difficulty sleeping	561 (57.2)	420 (42.8)
Lack of fetal movement	167 (17)	814 (83)

Considering the awareness of obstetric and abnormal signs and symptoms, less than half of the participants were fully aware of the abnormal obstetric signs. For the abnormal signs and symptoms during the first trimester, only 45.2% were fully aware of the symptoms. In the second trimester, 39.0% were fully aware of the symptoms. Only 30.5% of the participants were fully aware of the abnormal signs and symptoms in the third trimester (Table 5).

**Table 5:** Knowledge of obstetric and abnormal signs and symptoms

Abnormal signs and symptoms in the first trimester	N	%
Not aware	83	8.5
Partially aware	455	46.3
Fully aware	443	45.2
Abnormal signs and symptoms in the second trimester	N	%
Not aware	178	18.1
Partially aware	421	42.9
Fully aware	382	39.0
Abnormal signs and symptoms in the third trimester	N	%
Not aware	204	20.8
Partially aware	478	48.7
Fully aware	299	30.5

In the assessment of factors associated with increased awareness levels during the three stages of pregnancy, certain factors were found to be related to the level of awareness. A significant positive correlation exists between age and knowledge of abnormal symptoms during the first, second, and third trimesters (P=0.027, 0.041, and 0.006) (r=0.139, 0.105, and 0.146), indicating that older females have a better level of knowledge compared to younger participants. Furthermore, a significant positive correlation was found between the level of education of the participants (P=0.043); however, the education level is only significant in the awareness of abnormal symptoms during the first trimester, but no significant association was found between education level and the level of knowledge regarding abnormal symptoms during the second and third trimesters (r=0.22, 0.061, P=0.578, 0.603). Nationality was not associated with any of the knowledge categories (P=0.064, 0.691, 0.205). Additionally, marital status, employment status, economic status, and smoking status were also not associated with any of the knowledge categories as indicated by (P=0.079, 0.802, 0.411), (P=0.063, 0.690, 0.341), (P=0.134, 0.397, 0.512), and P=0.105, 0.598, 0.319), respectively. Additionally, marital status, economic status, and smoking status were not associated with any of the knowledge categories (Table 6).

**Table 6:** Factors associated with increased awareness level about abnormal symptoms during the first, second, and third trimesters *Significant when the p-value is lower than 0.05

		Abnormal signs during the first trimester			Abnormal signs during the first trimester			Abnormal signs during the first trimester		
		Not aware	Fully aware	p-value	Not aware	Fully aware	p-value	Not aware	Fully aware	p-value
Age (in years)	18-25									
	26-35			0.027*			0.041*			0.006*
	36-45									
Nationality	Saudi			0.064			0.691			0.205
	Non-Saudi									
Marital status	Single			0.079			0.802			0.411
	Married									
	Divorced									
	Windowed									
Education level	Primary			0.043*			0.578			0.603
	Intermediate									
	Secondary									
	Bachelor’s degree									
	Postgraduate									
Employment status	Employed			0.063			0.69			0.341
	Unemployed									
	Student									
	Retired									
Economic status	<5,000			0.134			0.397			0.512
	5000-10000									
	10000-15000									
	>15000									
Smoking status	Smoker			0.105			0.598			0.319
	Non-smoker									
	Ex-smoker									

## Discussion

It is a fact that women's bodies go through some changes during pregnancy, and these changes are usually normal but sometimes can be uncomfortable. Abnormal signs and symptoms may end with serious complications women could spare like morning sickness that may lead to hyperemesis gravidarum and make women uncomfortable and scared; therefore, good awareness is essential to guide women through this period safely.

This study’s main objective was to assess the level of awareness and knowledge about normal and abnormal signs and symptoms of pregnancy among women of childbearing age in Saudi Arabia. The study findings noted that the majority of women demonstrated a good level of knowledge about the normal signs and symptoms of pregnancy. The vast majority, 82% of the participants, reported that they knew mood swings were normal symptoms during pregnancy, 80.1% were aware of nausea, 75.9% knew about fatigue, 68.9% knew about fainting or dizziness, 67.4% and 67.1% knew of heartburn and vomiting, respectively, and 65.9% knew cravings or an extreme aversion to eating and vaginal discharge with no itching. This finding is similar to the results of a study conducted by Amasha in Jordan which found that more than half of the sampled women were aware that fainting/dizziness, nausea, abdominal cramps, fatigue, and mood swings were normal symptoms of pregnancy [16]. The findings are also in agreement with a study conducted by Emelianova et al. on 193 pregnant women in Canada which found that 67.4% of the women experienced nausea and vomiting [17]. A similar study by Quaresma et al. conducted in Portugal on 49 pregnant women reported that 71.4% and 91.7% of the women were aware that back pain at 12 weeks and 37 weeks were symptoms of pregnancy [18]. The findings established that persistent mood swings and persistent nausea were the most recognized abnormal symptoms during the first trimester, with back pain, stretch marks, and headache being the most known symptoms during the second trimester, while frequent urination and lower limb edema being the most known abnormal symptoms during the third trimester. These findings are consistent with the findings of the study conducted by Okour et al. among women in Jordan which found that the majority of the women recognized severe headache, back pain, and swelling of the lower limb as the symptoms of abnormal pregnancy [19].

On the other hand, considering the awareness of obstetric and abnormal signs and symptoms, less than half of the participants were fully aware of the abnormal obstetric signs. For the abnormal signs and symptoms during the first trimester, only 45.2% were fully aware of the symptoms. In the second trimester, 39.0% were fully aware of the symptoms. Only 30.5% of the participants were fully aware of the abnormal signs and symptoms in the third trimester. The reported finding is higher than the reported finding by Abu-Shaheen et al., in which it was reported that only 13.5% of the women were knowledgeable about the three complications during pregnancy followed by 3.4% who were able to identify three complications during labor and the postpartum period [20]. The findings might indicate inadequate education by the healthcare providers during the antenatal visits and other education programs. Therefore, teaching and emphasizing the abnormal signs and symptoms should be included in the health education program during the antenatal period. It is imperative to note that knowing abnormal signs and symptoms would not necessarily ensure that pregnant women could recognize the severity of the problem. Healthcare personnel should also inform all pregnant women to be aware of the abnormal signs as well as go to the hospital as soon as such abnormal symptoms occur.

The study established a statistically significant relationship between age and knowledge of abnormal symptoms during the first, second, and third trimesters (P=0.027, 0.041, and 0.006) (r=0.139, 0.105, and 0.146) indicating that older females have a better level of knowledge compared to younger participants. Furthermore, a statistically significant relationship was found between the level of education of the participants and the level of awareness of abnormal symptoms during the first trimester (P=0.043). However, there was no significant relationship that was found between education level and the level of knowledge regarding abnormal symptoms during the second and third trimesters (r=0.22, 0.061, P=0.578, 0.603). The study findings suggest that age and level of education were significantly associated with the level of knowledge of abnormal signs and symptoms at different stages of pregnancy. In regards to age, females above 26 years of age had better awareness of abnormal signs and symptoms than the younger age. This finding is consistent with Chortatos et al. in which it was reported that females older than 25 years had better awareness of obstetric abnormalities and complications during pregnancy [21]. Considering the education level, 95.2% of the participants had attained secondary education and above; hence, they had a good understanding and knowledge of normal and abnormal signs and symptoms of pregnancy. This was shown to be positively correlated with the level of awareness of abnormal symptoms during the first trimester.

Limitations

The main limitation of this study is that data were collected via a self-reported survey through online networks which may be influenced by reporting bias.

Recommendations

We recommend that more research should be conducted to measure women's awareness about the normal and abnormal symptoms of pregnancy, and more awareness programs should be carried out in the region as a way of enhancing a better understanding of normal and abnormal pregnancy symptoms in order to aid them through their pregnancy and make them more comfortable.

## Conclusions

The findings in this study indicated that the majority of the women had a good level of awareness of normal signs and symptoms of pregnancy, while less than half of the women were fully aware of the abnormal signs and symptoms at different stages of pregnancy. The older respondents and people with higher levels of education demonstrated more knowledge. The two variables, age and education level, had a statistically significant relationship with the knowledge of abnormal signs and symptoms of pregnancy with education level being only significant during the first trimester period. Based on the findings of this study, women experienced different signs and symptoms during pregnancy, and many of them are clear and others are unclear. A high knowledge of the signs and symptoms of pregnancy is crucial to women of reproductive age to aid them through pregnancy and make them comfortable.

## Appendices

Appendix A

Consent Form

The aim of this research was to evaluate the level of awareness and knowledge about positive signs of pregnancy among women of childbearing age in Saudi Arabia.

The information in this questionnaire is very confidential and will be used for the purpose of scientific research and educational benefit only.

May we ask you a few questions?

-Yes

-No

For inquiries and communication with the research team, please contact the principal investigator: Dr. Taghreed Basri.

Email:

Appendix B

Questionnaire

Dear Mother:

Hello, I am a health practitioner in the Health Cluster in Madinah. I am conducting a study on the extent of mothers’ awareness of the normal and abnormal signs of pregnancy given that the delay in detecting abnormal symptoms exposes the mother and fetus to complications.

I would like to know your experiences and information about pregnancy symptoms, which have a positive impact on developing an educational plan for mothers. To keep you and your baby safe, we kindly ask you to complete this questionnaire. Your responses are anonymous and you can skip any questions you feel uncomfortable with. Thank you for your participation.

**Table 7:** Questionnaire

Sociodemographic and obstetrical history				
Are you living in KSA?				
		Yes	No	
Nationality?				
		Saudi	Not Saudi	
Age?				
Less than 18	18-30	31-40	41-50	More than 50
Educational level?				
Primary school	Intermediate school	High school	University	Postgraduate
Employment status?				
	Student	Employed	Retired	Not employed
Economic status?				
		Under 5000 SR	Between 5000 and 15000 SR	Over 15000 SR
Smoking status?				
	Smoker	Non-smoker	Ex-smoker	Others (mention…..)
Do you have any chronic disease?				
Hypertension	Diabetes mellitus	Asthma	Thyroid problems	None
Blood group?				
AB+	A+	B+	O+	
AB-	A-	B-	O-	I don’t know
No. of pregnancies?				
	Primigravida	1-3 times	4-5 times	≥6 times
No. of abortions?				
	None	<3 times	≥3 times	
Current gestational age?				
	Not pregnant	1^st ^trimester	2^nd^ trimester	3^rd^ trimester
Number of children?				
	None	01-Mar	04-Jun	≥7
If you’re pregnant now or have previously, how did know?				
	By home urine pregnancy test	By blood test in a hospital	By Ultrasonography at a hospital	By experience from previous pregnancy\ies
Are you using a folic acid supplement?				
		Yes	No	
1st trimester symptoms				
Do you know whether the following are normal/abnormal symptoms and signs of pregnancy?				
Fainting/dizziness?				
		Yes	No	
Nausea?				
		Yes	No	
Vomiting?				
		Yes	No	
Fatiguability?				
		Yes	No	
Heartburn?				
		Yes	No	
Mood swings?				
		Yes	No	
Extreme cravings or aversion to eating?				
		Yes	No	
Swelling or pain in the breasts?				
		Yes	No	
Constipation?				
		Yes	No	
Vaginal discharge?				
		Yes	No	
Abdominal cramps?				
		Yes	No	
2nd trimester symptoms				
Do you know whether the following are normal/abnormal symptoms and signs of pregnancy?				
Do these (above) symptoms still persist?				
		Yes	No	
Heartburn or regurgitation?				
		Yes	No	
Stretch marks?				
		Yes	No	
Breast engorgement?				
		Yes	No	
Breast pain?				
		Yes	No	
Swollen gums or painful gums?				
		Yes	No	
Nosebleeds?				
		Yes	No	
Pain at a specific site on the abdomen?				
		Yes	No	
Headache?				
		Yes	No	
Blurry vision?				
		Yes	No	
Backache?				
		Yes	No	
Leg’s muscles cramps?				
		Yes	No	
Fever/febrile?				
		Yes	No	
Using iron or calcium supplements?				
		Yes	No	
Burning sensation during urination?				
		Yes	No	
Previous diagnosis of urinary infection?				
		Yes	No	
Any abnormal discharge/s and its/their color?				
		Yes, color is……….	No	
Is/are it/they smelly?				
		Yes	No	
Exaggerated smell sensation?				
		Yes	No	
Nasal sensitivity and sneezing?				
		Yes	No	
Persist pregnancy craving or stopped?				
		Persist	Stopped	
3rd trimester symptoms				
Do you know whether the following is a normal/abnormal symptoms and sign of pregnancy?				
Do these (above) symptoms still persist?				
		Yes	No	
Lower limbs edema				
		Yes	No	
Severe abdominal pain				
		Yes	No	
Brownish discharge				
		Yes	No	
False labor				
		Yes	No	
Hemorrhoids				
		Yes	No	
Varicose veins				
		Yes	No	
Frequent urination				
		Yes	No	
Urine incontinence				
		Yes	No	
Rapid gain weight				
		Yes	No	
Constipation				
		Yes	No	
Shortness of breath				
		Yes	No	
Difficulty sleeping				
		Yes	No	
Reduce fetal movement				
		Yes	No	
